# Supplementary material for: Spatial distribution and determinants of HIV high burden in the Southern African sub-region
Source: PLoS One. 2024 Apr 26;19(4):e0301850. doi: 10.1371/journal.pone.0301850 (PMC11051620; doi:10.1371/journal.pone.0301850)
Supplement: S1 File — (DOCX) [file pone.0301850.s001.docx]

**SUPPLEMENTARY FILE 1**

***Table 1. Factors associated with HIV positivity identified by logistics regression (Malawi)***

| **Variables** |  | **Crude odds ratio** | **95% CI** | **Adjusted odds ratio** | **95% CI** |
| --- | --- | --- | --- | --- | --- |
| ***Age*** |  |  |  |  |  |
|  | 15-24 years | Reference |  | Reference |  |
|  | 25-34 years | 2.43 | 2.13 – 2.79* | 1.87 | 1.57 – 2.38* |
|  | 35+ years | 3.94 | 3.46 – 4.47* | 3.11 | 2.60 – 3.73* |
| ***Education*** |  |  |  |  |  |
|  | No formal education | Reference |  | Reference |  |
|  | Primary | 0.67 | 0.57 – 0.79* | 0.96 | 0.80 – 1.17 |
|  | Secondary/Higher | 0.74 | 0.62 – 0.88* | 0.98 | 0.79 – 1.22 |
| ***Marital status*** | | | | | |
|  | Never married | Reference |  | Reference |  |
|  | Married/Living together | 2.52 | 2.19 – 2.90* | 1.73 | 1.34 – 2.24* |
|  | Separated/Widow | 5.24 | 4.38 – 6.27* | 2.57 | 1.89 – 3.48* |
| ***Wealth index*** | | | | | |
|  | Poor | Reference |  | Reference |  |
|  | Middle | 1.05 | 0.91 – 1.22 | 1.07 | 0.90 – 1.26 |
|  | Rich | 1.24 | 1.11 – 1.39* | 1.09 | 0.94 – 1.26 |
| ***Employment status*** | | | | | |
|  | None | Reference |  | Reference |  |
|  | Employed | 1.14 | 1.02 – 1.28* | 0.74 | 0.64 – 0.84* |
| ***Gender*** | | | | | |
|  | Female | Reference |  | Reference |  |
|  | Male | 0.78 | 0.70 – 0.86* | 0.91 | 0.80 – 1.03 |
| ***Residence*** | | | | | |
|  | Urban | Reference |  | Reference |  |
|  | Rural | 0.64 | 0.58 – 0.72* | 0.62 | 0.53 – 0.71* |
| ***Sexually transmitted infections in the last 12 months*** | | | | | |
|  | No | Reference |  | Reference |  |
|  | Yes | 2.46 | 1.91 – 3.16* | 2.00 | 1.51 – 2.65* |
|  | Not sure | 0.80 | 0.25 – 2.25 | 1.21 | 0.42 – 3.50 |
| ***Ever tested for HIV*** | | | | |  |
|  | No | Reference |  | Reference |  |
|  | Yes | 2.17 | 1.87 – 2.51* | 1.23 | 1.02 – 1.49 |
| ***Used condom every time had sex with most recent partner in last 12 months*** | | | | | |
|  | No | Reference |  | Reference |  |
|  | Yes | 1.10 | 0.96 – 1.26 | 1.73 | 1.47 – 2.04* |

***Table 2. Factors associated with HIV positivity identified by logistics regression (Mozambique)***

| **Variables** |  | **Crude odds ratio** | **95% CI** | **Adjusted odds ratio** | **95% CI** |  |
| --- | --- | --- | --- | --- | --- | --- |
| ***Age*** |  |  |  |  |  |  |
|  | 15-24 years | Reference |  | Reference |  |  |
|  | 25-34 years | 2.84 | 2.45 – 3.28* | 2.09 | 1.75 – 2.50* |  |
|  | 35+ years | 3.00 | 2.62 – 3.44* | 2.33 | 1.95 – 2.79* |  |
| ***Education*** |  |  |  |  |  |  |
|  | No formal education | Reference |  | Reference |  |  |
|  | Primary | 1.14 | 0.99 – 1.31 | 1.20 | 1.00 – 1.43 |  |
|  | Secondary/Higher | 0.87 | 0.75 – 1.02 | 0.81 | 0.65 – 1.01 |  |
| ***Marital status*** | | | | | |  |
|  | Never married | Reference |  | Reference |  |  |
|  | Married/Living together | 2.17 | 1.85 – 2.55* | 1.15 | 0.92 – 1.45 |  |
|  | Separated/Widow | 5.71 | 4.75 – 6.84* | 2.88 | 2.23 – 3.72* |  |
| ***Wealth index*** | | | | | |  |
|  | Poor | Reference |  | Reference |  |  |
|  | Middle | 1.46 | 1.22 – 1.73* | 1.46 | 1.20 – 1.79* |  |
|  | Rich | 2.08 | 1.82 – 2.37* | 2.23 | 1.85 – 2.68* |  |
| ***Employment status*** | | | | | |  |
|  | None | Reference |  | Reference |  |  |
|  | Employed | 1.27 | 1.15 – 1.41* | 0.97 | 0.85 – 1.11 |  |
| ***Gender*** | | | | | |  |
|  | Female | Reference |  | Reference |  |  |
|  | Male | 0.60 | 0.54 – 0.68* | 0.79 | 0.68 – 0.90* |  |
| ***Residence*** | | | | | |  |
|  | Urban | Reference |  | Reference |  |  |
|  | Rural | 0.68 | 0.61 – 0.75* | 0.96 | 0.83 – 1.12 |  |
| ***Sexually transmitted infections in the last 12 months*** | | | | | |  |
|  | No | Reference |  | Reference |  |  |
|  | Yes | 2.06 | 1.67 – 2.55* | 1.94 | 1.53 – 2.46* |  |
|  | Not sure | 1.97 | 1.05 – 3.70 | 3.15 | 1.60 – 6.19* |  |
| ***Ever tested for HIV*** | | | | |  |  |
|  | No | Reference |  | Reference |  |  |
|  | Yes | 2.17 | 1.87 – 2.51* | 1.70 | 1.47 – 1.97* |  |
| ***Used condom every time had sex with most recent partner in last 12 months*** | | | | | |  |
|  | No | Reference |  | Reference |  |  |
|  | Yes | 1.25 | 1.09 – 1.45* | 1.33 | 1.12 – 1.59* |  |

***Table 3. Factors associated with HIV positivity identified by logistics regression (Namibia)***

| **Variables** |  | **Crude odds ratio** | **95% CI** | **Adjusted odds ratio** | **95% CI** |
| --- | --- | --- | --- | --- | --- |
| ***Age*** |  |  |  |  |  |
|  | 15-24 years | Reference |  | Reference |  |
|  | 25-34 years | 4.95 | 4.01 – 6.11* | 3.38 | 2.62 – 4.36* |
|  | 35+ years | 6.11 | 5.02 – 7.44* | 4.96 | 3.82 – 6.44* |
| ***Education*** |  |  |  |  |  |
|  | No formal education | Reference |  | Reference |  |
|  | Primary | 1.10 | 0.89 – 1.35 | 1.15 | 0.89 – 1.49 |
|  | Secondary/Higher | 0.66 | 0.55 – 0.80* | 0.88 | 0.68 – 1.14 |
| ***Marital status*** | | | | | |
|  | Never married | Reference |  | Reference |  |
|  | Married/Living together | 1.56 | 1.37 – 1.78* | 1.03 | 0.86 – 1.24 |
|  | Separated/Widow | 3.22 | 2.67 – 3.89* | 1.32 | 0.97 – 1.79 |
| ***Wealth index*** | | | | | |
|  | Poor | Reference |  | Reference |  |
|  | Middle | 0.87 | 0.75 – 1.02 | 0.76 | 0.63 – 0.92* |
|  | Rich | 0.50 | 0.43 – 0.57* | 0.37 | 0.30 – 0.46* |
| ***Employment status*** | | | | | |
|  | None | Reference |  | Reference |  |
|  | Employed | 1.47 | 1.30 – 1.66* | 1.14 | 0.97 – 1.34 |
| ***Gender*** | | | | | |
|  | Female | Reference |  | Reference |  |
|  | Male | 0.62 | 0.55 – 0.70* | 0.66 | 0.56 – 0.77* |
| ***Residence*** | | | | | |
|  | Urban | Reference |  | Reference |  |
|  | Rural | 1.10 | 0.97 – 1.24 | 0.77 | 0.65 – 0.91* |
| ***Sexually transmitted infections in the last 12 months*** | | | | | |
|  | No | Reference |  | Reference |  |
|  | Yes | 1.98 | 1.50 – 2.61* | 1.77 | 1.28 – 2.46* |
|  | Not sure | 2.38 | 0.63 – 8.99 | 1.20 | 0.23 – 6.35 |
| ***Ever tested for HIV*** | | | | |  |
|  | No | Reference |  | Reference |  |
|  | Yes | 4.41 | 3.61 – 5.38* | 2.57 | 1.98 – 3.34* |
| ***Used condom every time had sex with most recent partner in last 12 months*** | | | | | |
|  | No | Reference |  | Reference |  |
|  | Yes | 1.46 | 1.27 – 1.67* | 2.04 | 1.73 – 2.40* |

***Table 4. Factors associated with HIV positivity identified by logistics regression (South Africa)***

| **Variables** |  | **Crude odds ratio** | **95% CI** | **Adjusted odds ratio** | **95% CI** |
| --- | --- | --- | --- | --- | --- |
| ***Age*** |  |  |  |  |  |
|  | 15-24 years | Reference |  | Reference |  |
|  | 25-34 years | 4.27 | 3.48 – 5.22* | 3.68 | 2.88 – 4.69* |
|  | 35+ years | 5.02 | 4.14 – 6.10* | 5.01 | 3.87 – 6.50* |
| ***Education*** |  |  |  |  |  |
|  | No formal education | Reference |  | Reference |  |
|  | Primary | 1.11 | 0.75 – 1.65 | 1.41 | 0.87 – 2.28 |
|  | Secondary/Higher | 0.74 | 0.51 – 1.07 | 0.06 | 0.64 – 1.61 |
| ***Marital status*** | | | | | |
|  | Never married | Reference |  | Reference |  |
|  | Married/Living together | 1.47 | 1.27 – 1.70* | 1.03 | 0.85 – 1.24 |
|  | Separated/Widow | 3.15 | 2.44 – 4.06* | 1.61 | 1.15 – 2.23* |
| ***Wealth index*** | | | | | |
|  | Poor | Reference |  | Reference |  |
|  | Middle | 0.85 | 0.72 – 0.99 | 0.74 | 0.61 – 0.91* |
|  | Rich | 0.56 | 0.48 – 0.67* | 0.43 | 0.34 – 0.54* |
| ***Employment status*** | | | | | |
|  | None | Reference |  | Reference |  |
|  | Employed | 1.20 | 1.05 – 1.39* | 0.89 | 0.75 – 1.05 |
| ***Gender*** | | | | | |
|  | Female | Reference |  | Reference |  |
|  | Male | 0.44 | 0.38 – 0.51* | 0.43 | 0.36 – 0.51* |
| ***Residence*** | | | | | |
|  | Urban | Reference |  | Reference |  |
|  | Rural | 0.99 | 0.87 – 1.13 | 0.79 | 0.66 – 0.94* |
| ***Sexually transmitted infections in the last 12 months*** | | | | | |
|  | No | Reference |  | Reference |  |
|  | Yes | 2.16 | 1.65 – 2.84* | 1.98 | 1.44 – 2.72* |
|  | Not sure |  |  |  |  |
| ***Ever tested for HIV*** | | | | |  |
|  | No | Reference |  | Reference |  |
|  | Yes | 1.46 | 1.26 – 1.69* | 1.38 | 1.06 – 1.79* |
| ***Used condom every time had sex with most recent partner in last 12 months*** | | | | | |
|  | No | Reference |  | Reference |  |
|  | Yes | 2.73 | 2.21 – 3.36* | 2.01 | 1.70 – 2.39 |

***Table 5. Factors associated with HIV positivity identified by logistics regression (Zambia)***

| **Variables** |  | **Crude odds ratio** | **95% CI** | **Adjusted odds ratio** | **95% CI** |
| --- | --- | --- | --- | --- | --- |
| ***Age*** |  |  |  |  |  |
|  | 15-24 years | Reference |  | Reference |  |
|  | 25-34 years | 3.88 | 3.43 – 4.39* | 2.44 | 2.08 – 2.86* |
|  | 35+ years | 6.10 | 5.43 – 6.84* | 4.66 | 3.95 – 5.50* |
| ***Education*** |  |  |  |  |  |
|  | No formal education | Reference |  | Reference |  |
|  | Primary | 1.12 | 0.94 – 1.34 | 1.36 | 1.10 – 1.68* |
|  | Secondary/Higher | 1.27 | 1.06 – 1.51 | 1.44 | 1.16 – 1.80* |
| ***Marital status*** | | | | | |
|  | Never married | Reference |  | Reference |  |
|  | Married/Living together | 2.85 | 2.55 – 3.17* | 1.32 | 1.11 – 1.56* |
|  | Separated/Widow | 8.70 | 7.62 – 9.93* | 3.00 | 2.46 – 3.67* |
| ***Wealth index*** | | | | | |
|  | Poor | Reference |  | Reference |  |
|  | Middle | 1.63 | 1.45 – 1.83* | 1.38 | 1.20 – 1.58* |
|  | Rich | 2.30 | 2.09 – 2.51* | 1.52 | 1.30 – 1.78* |
| ***Employment status*** | | | | | |
|  | None | Reference |  | Reference |  |
|  | Employed | 1.46 | 1.35 – 1.59* | 0.96 | 0.86 – 1.07 |
| ***Gender*** | | | | | |
|  | Female | Reference |  | Reference |  |
|  | Male | 0.59 | 0.54 – 0.64* | 0.56 | 0.50 – 0.62* |
| ***Residence*** | | | | | |
|  | Urban | Reference |  | Reference |  |
|  | Rural | 0.43 | 0.40 – 0.47* | 0.55 | 0.49 – 0.63* |
| ***Sexually transmitted infections in the last 12 months*** | | | | | |
|  | No | Reference |  | Reference |  |
|  | Yes | 2.28 | 1.89 – 2.75* | 2.42 | 1.96 – 2.99* |
|  | Not sure | 2.69 | 0.28 – 25.82 | 4.05 | 0.36 – 45.13 |
| ***Ever tested for HIV*** | | | | |  |
|  | No | Reference |  | Reference |  |
|  | Yes | 3.78 | 3.22 – 4.43* | 1.49 | 1.22 – 1.83* |
| ***Used condom every time had sex with most recent partner in last 12 months*** | | | | | |
|  | No | Reference |  | Reference |  |
|  | Yes | 1.77 | 1.61 – 1.96* | 2.12 | 1.88 – 2.39* |

***Table 6. Factors associated with HIV positivity identified by logistics regression (Zimbabwe)***

| **Variables** |  | **Crude odds ratio** | **95% CI** | **Adjusted odds ratio** | **95% CI** |
| --- | --- | --- | --- | --- | --- |
| ***Age*** |  |  |  |  |  |
|  | 15-24 years | Reference |  | Reference |  |
|  | 25-34 years | 3.60 | 3.16 – 4.11* | 2.33 | 1.96 – 2.77* |
|  | 35+ years | 6.83 | 6.03 – 7.74* | 4.20 | 3.52 – 5.02* |
| ***Education*** |  |  |  |  |  |
|  | No formal education | Reference |  | Reference |  |
|  | Primary | 1.34 | 0.84 – 2.14 | 3.17 | 1.64 – 6.16* |
|  | Secondary/Higher | 0.95 | 0.59 – 1.51 | 2.51 | 1.30 – 4.85* |
| ***Marital status*** | | | | | |
|  | Never married | Reference |  | Reference |  |
|  | Married/Living together | 3.81 | 3.35 – 4.34* | 2.98 | 2.37 – 3.73* |
|  | Separated/Widow | 11.58 | 9.91 – 13.53* | 3.62 | 2.83 – 4.64* |
| ***Wealth index*** | | | | | |
|  | Poor | Reference |  | Reference |  |
|  | Middle | 0.98 | 0.87 – 1.12 | 1.10 | 0.93 – 1.28 |
|  | Rich | 0.95 | 0.86 – 1.04 | 0.83 | 0.68 – 1.02 |
| ***Employment status*** | | | | | |
|  | None | Reference |  | Reference |  |
|  | Employed | 1.28 | 1.18 – 1.40* | 0.83 | 0.74 – 0.93* |
| ***Gender*** | | | | | |
|  | Female | Reference |  | Reference |  |
|  | Male | 0.64 | 0.59 – 0.70* | 0.72 | 0.64 – 0.81* |
| ***Residence*** | | | | | |
|  | Urban | Reference |  | Reference |  |
|  | Rural | 0.93 | 0.85 – 1.01 | 0.80 | 0.66 – 0.97 |
| ***Sexually transmitted infections in the last 12 months*** | | | | | |
|  | No | Reference |  | Reference |  |
|  | Yes | 3.47 | 2.78 – 4.33* | 2.52 | 1.92 – 3.30* |
|  | Not sure | 1.96 | 0.71 – 5.40 | 2.22 | 0.73 – 6.70 |
| ***Ever tested for HIV*** | | | | |  |
|  | No | Reference |  | Reference |  |
|  | Yes | 3.46 | 3.02 – 3.97* | 1.49 | 1.24 – 1.77* |
| ***Used condom every time had sex with most recent partner in last 12 months*** | | | | | |
|  | No | Reference |  | Reference |  |
|  | Yes | 3.12 | 2.83 – 3.45* | 5.14 | 4.53 – 5.84* |

* p<0.05
