# Supplementary material for: Spatial distribution and determinants of HIV high burden in the Southern African sub-region
Source: PLoS One. 2024 Apr 26;19(4):e0301850. doi: 10.1371/journal.pone.0301850 (PMC11051620; doi:10.1371/journal.pone.0301850)
Supplement: S2 File — Local Spatial Autocorrelation cluster maps. (DOCX) [file pone.0301850.s002.docx]

**SUPPLEMENTARY FILE 2**


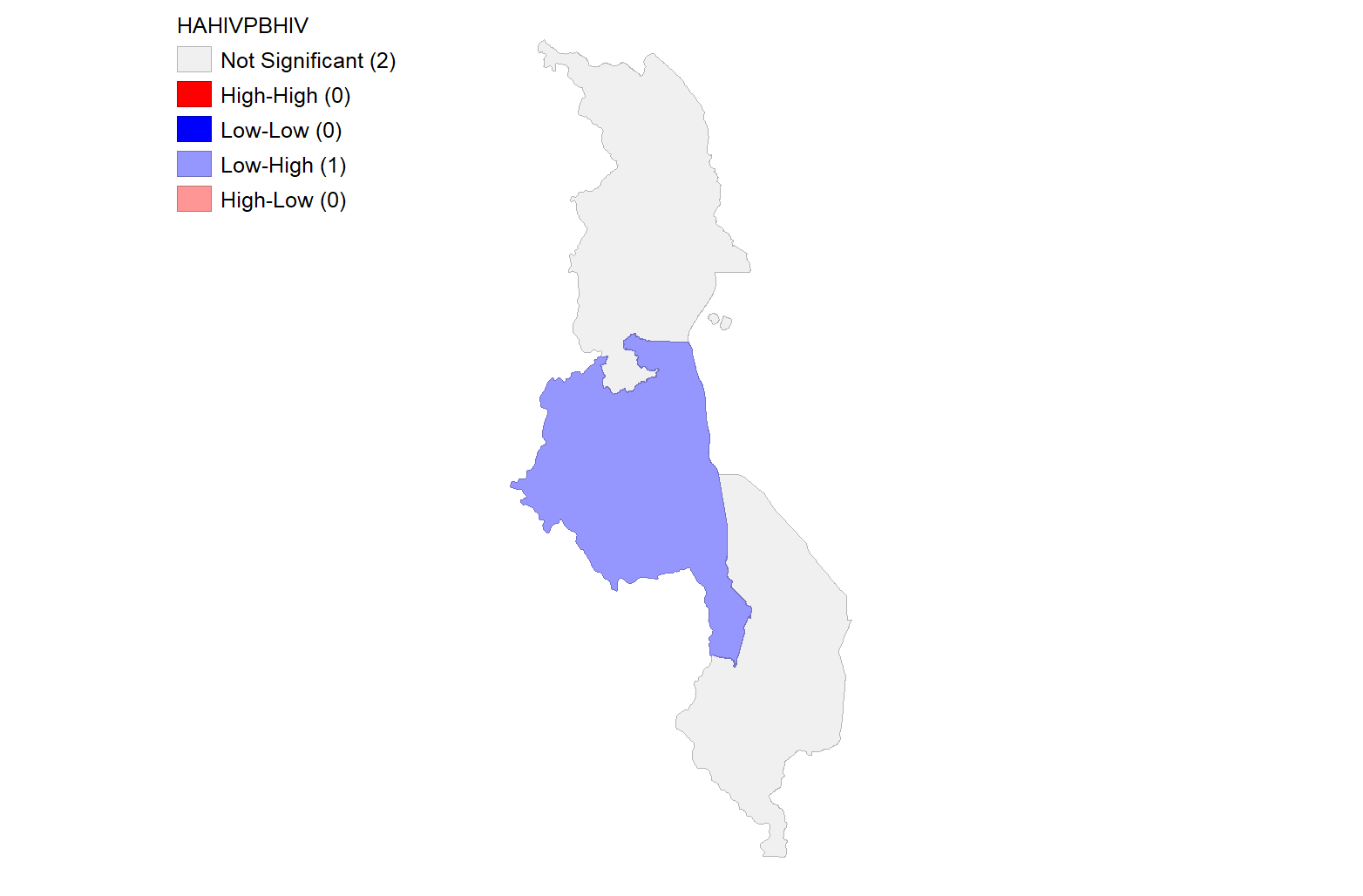


***Fig. 1*** *shows* *the Local Spatial Autocorrelation statistically significant cluster map. The study shows a Low-High HIV occurrence and cluster around the Central Region of Malawi.*


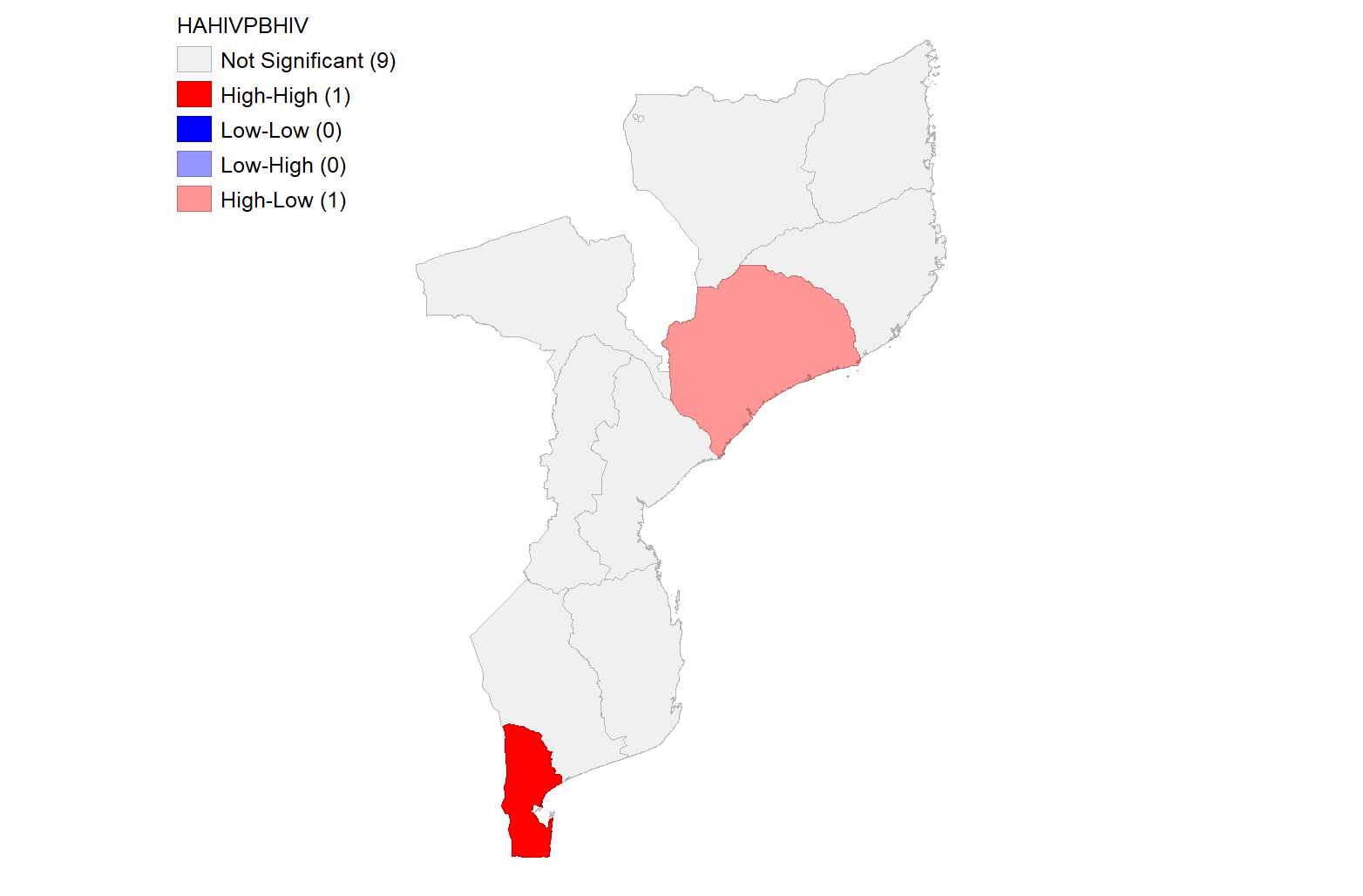


***Fig. 2*** *shows* *the Local Spatial Autocorrelation statistically significant cluster map. The study shows High-Low cluster in Zambezia Province and the High-High cluster in Maputo Province of Mozambique.*


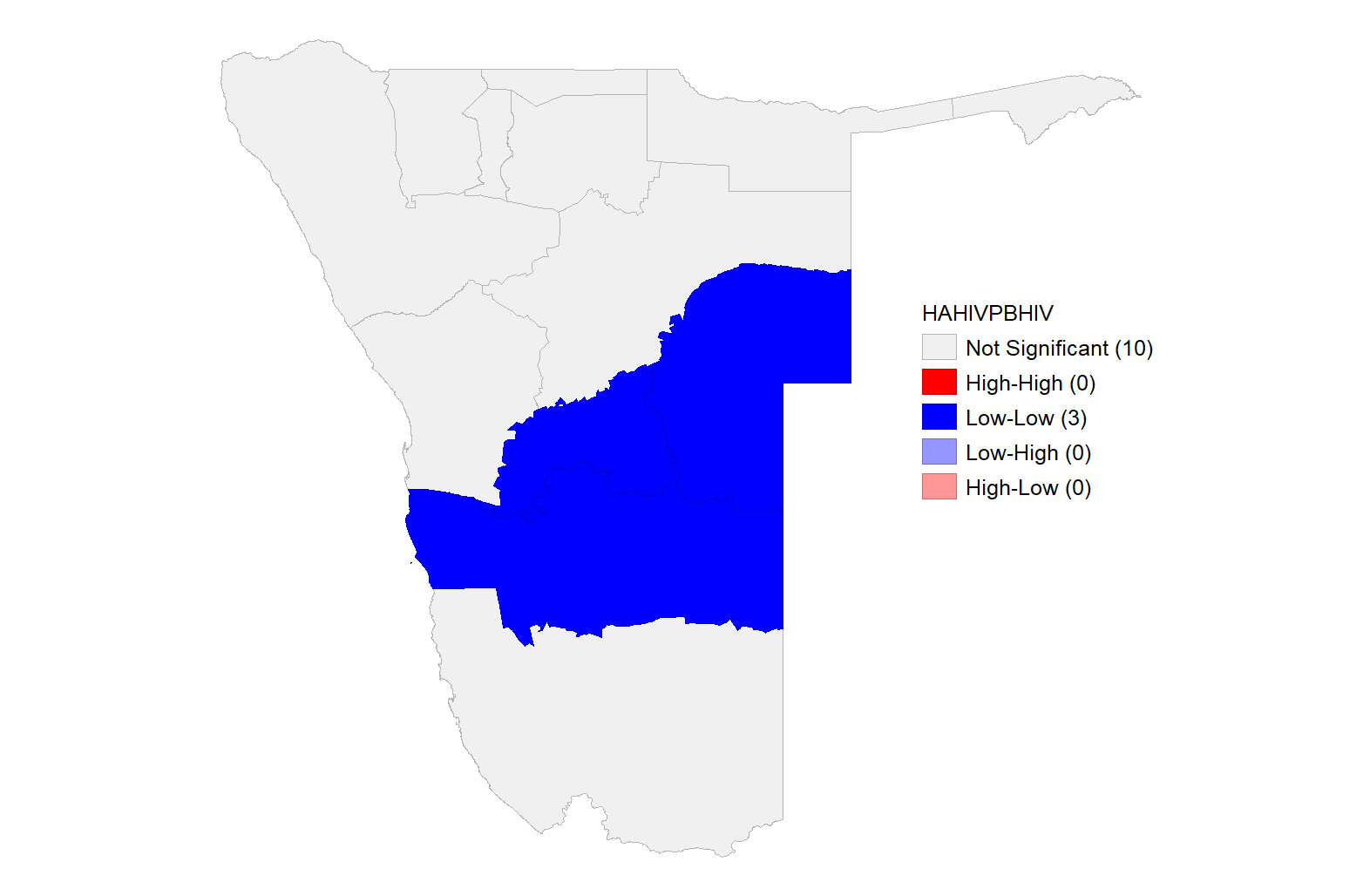


***Fig. 3*** *shows* *the Local Spatial Autocorrelation statistically significant cluster map. The study shows there were also Low-Low clusters in Omaheke, Khomas and Hardap Regions of Namibia.*


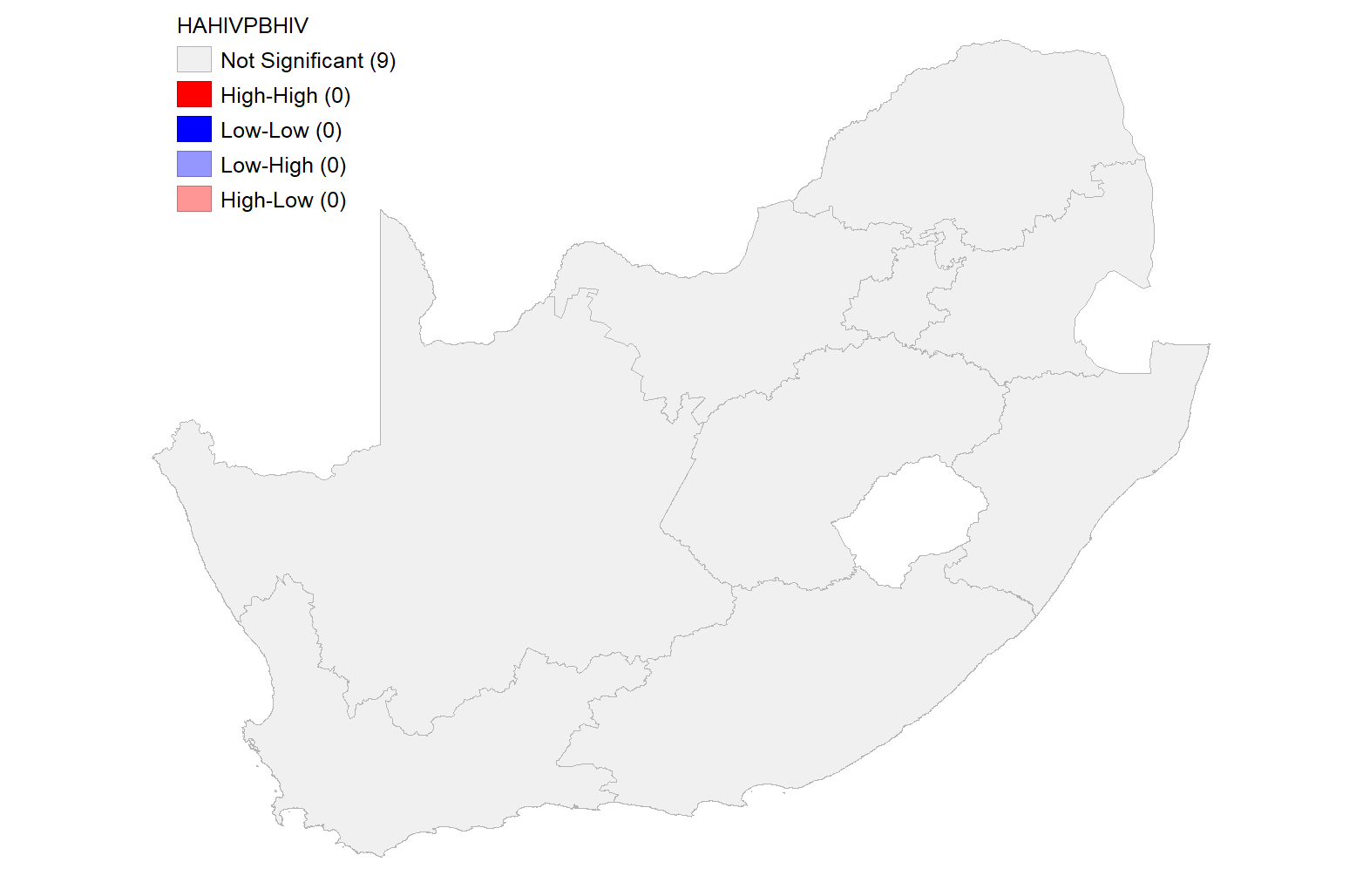


***Fig. 4*** *shows* *the Local Spatial Autocorrelation cluster map. There was no significant clustering among the South African Provinces.*


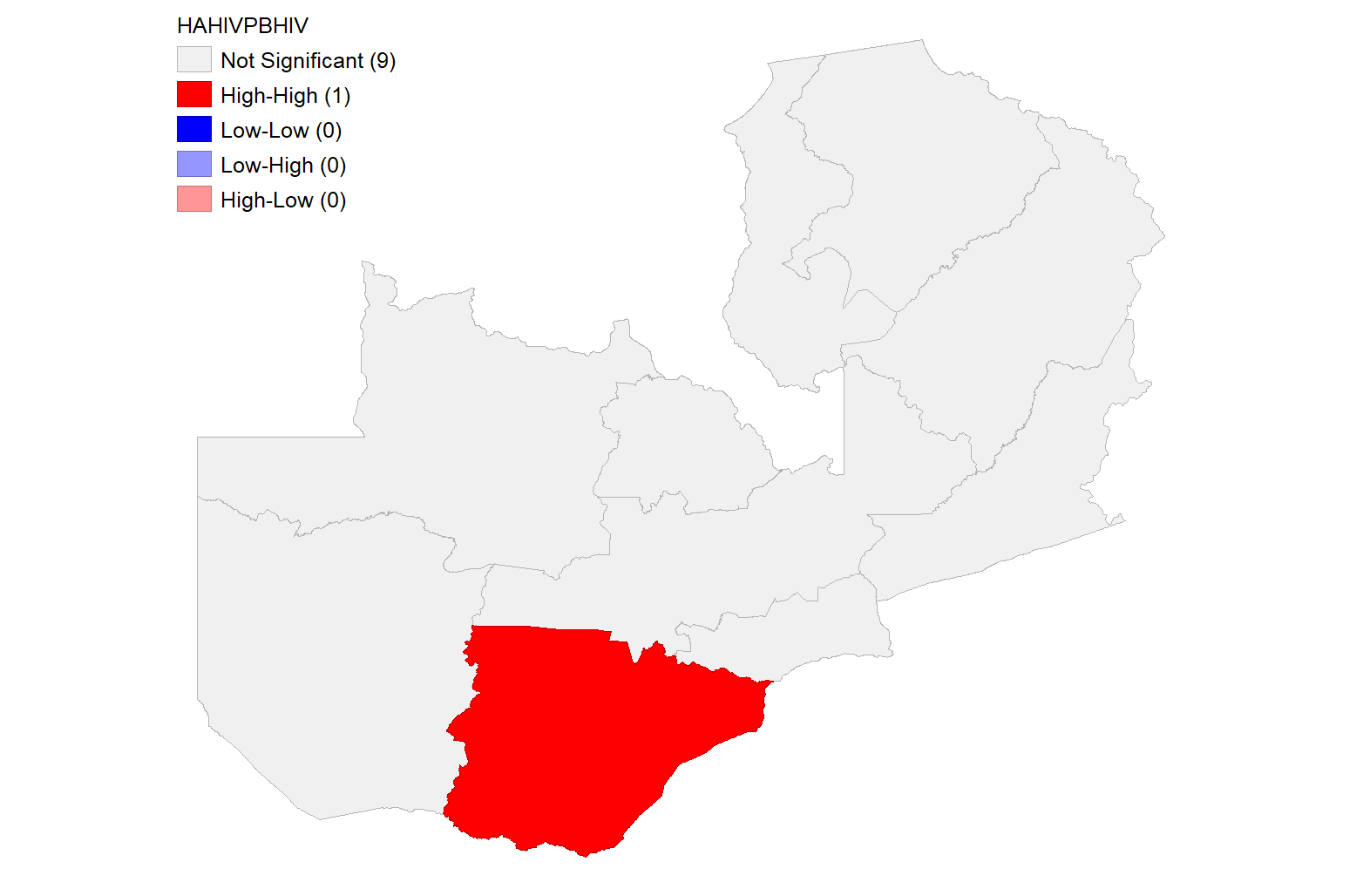


***Fig. 5*** *shows* *the Local Spatial Autocorrelation statistically significant cluster map. The study shows High-High cluster in Southern Province of Zambia.*


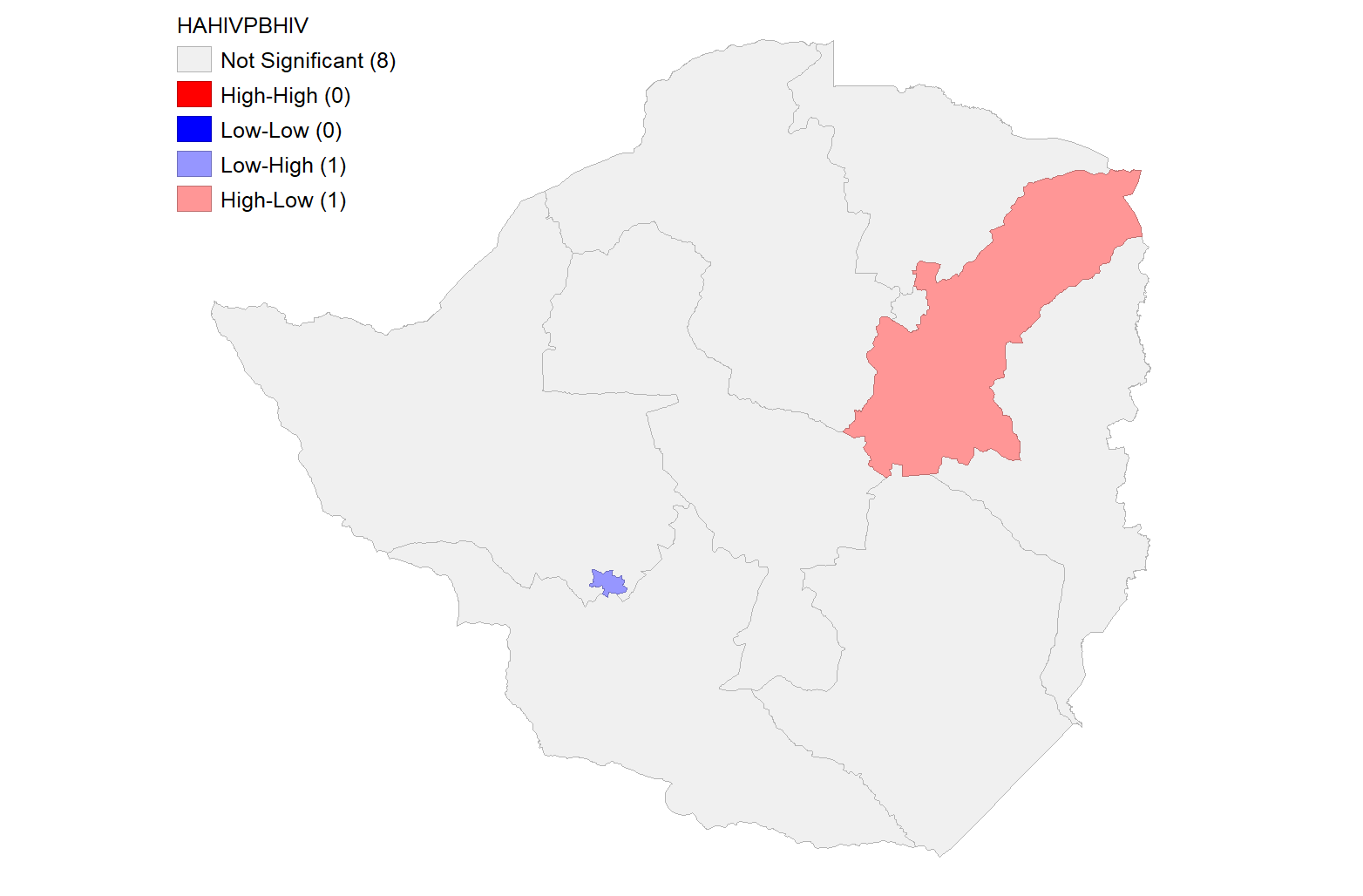


***Fig. 6*** *shows* *the Local Spatial Autocorrelation statistically significant cluster map. The study shows Low-High cluster in Bulawayo Province and High-Low cluster in Mashonaland East Province of Zimbabwe.*
