# Supplementary material for: Spatial distribution and determinants of HIV high burden in the Southern African sub-region
Source: PLoS One. 2024 Apr 26;19(4):e0301850. doi: 10.1371/journal.pone.0301850 (PMC11051620; doi:10.1371/journal.pone.0301850)
Supplement: Attachment — Submitted filename: Response to reviewers_Spatial Distribution_Revision.docx [file pone.0301850.s003.docx]

**Response to reviewers**

Reviewer #1: Dear Editor,
1. Thank you for inviting me to review this interesting manuscript.
The authors are addressing HIV, an epidemic whose incidence and prevalence in Southern Africa has remained high despite various interventions in the past 4 decades. Their research aimed at further evaluating the drivers of this epidemic in Southern Africa is relevant. While national figures are relevant, they may fall short of revealing the specific geographical areas and populations with high rates of HIV as well as the targetable risk factors specific to these populations and settings. Hence this manuscript, arising from secondary data analysis with timeseries and spatial autocorrelation methods, in which the authors have attempted to bridge this gap is worthy of publication in your prestigious journal.

The abstract is well written and gives a succinct account of the research question and the main results of the study. Although brief, the literature review reveals the main risk factors of HIV in the region and the gap in knowledge that still need s to be addressed. The authors have used appropriate research methods and analysis of the data. Generally, the data is well presented in scientific tables and figures. The discussion is coherent and well written. The conclusions are appropriate and relevant study limitations were highlighted.

***Response****: Thank you for the comments.*

2. The target audience may receive the authors’ message with better clarity if the following issues are considered:
• The data in Table 4 (HIV prevalence univariate local Moran’s I among selected Southern African countries) is scanty. The authors should add the columns with z-values and P values.

***Response:*** *Thank you for the advice. We have created new columns for the Z and p values.*

3. • The country specific autocorrelation figures (S1_6 figures) look beautiful, however, only the hot spots can be discerned also with some difficulty. The cold spots are not easy to see. The geographical regions of various countries, apart from the cities, are also not labelled on the maps yet in the discussion the authors regularly refer to them. I propose that the authors easily visible maps with clearer colour distinctions according to the different HIV prevalence and appropriately label the relevant provinces/geographical locations that were compared during data analysis.

***Response:*** *Thank for the comments, some of the hot and cold spots has been labelled.*

4. • One of the key results is that urbanization is a common feature associated with clustering of high HIV prevalence areas in all the six countries. However, there could be a few peculiar drivers of the epidemic in some urban areas that are not generalizable. A case in point is Rustenburg which is a mining town in South Africa. The authors could further strengthen the message in the manuscript by identifying and discussing the possible drivers of HIV in the peculiar hot spots in the various countries.

***Response:*** *Thank you for raising this issue and giving an example. We have identified some of the driving factors for some of the urban areas.*

5. • There is a noticeable trend in the reduction in the prevalence of HIV all the other countries except South Africa. In the discussion, the authors have alluded to the possible interventions that worked. A brief elaboration of these would make their point clearer to the audience. Secondly, it will be quite interesting if the authors discuss how the successful interventions relate to the spatial clustering observed in the current study. After reading the nice discussion thus far, the audience is left with some questions:
o Did the various countries use different or similar strategies for the prevention of HIV? Could there be country specific factors that hinder the successful outcome of similar interventions in South Africa?

***Response:*** *Thank you for the comments and suggestions. There were multiple and similar interventions across the countries. They were all part of the US PEPFAR programs. South Africa prevalence trend was not of failure per see despite the increase in annual average prevalence rate. The country has a good proportion of patients on ART and viral suppression; however, the issue is the huge number of new HIV infections constantly increases or stagnates the burden on yearly basis.*

6. • In the discussion the authors stated that a poor wealth index was associated with the increased risk of HIV infection. The data in table 3 to an ordinary reader seems to suggest that participants in the middle and higher wealth index bracket had higher prevalence of HIV. Secondly in the country specific multivariate logistic regressions (Supplementary file S1) a poor wealth index was an independent risk factor for HIV in only Malawi, Namibia and South Africa. The authors need to further discuss this point so as to better explain their conclusion in view of the findings as indicated in the data.

***Response****: Thank you for the suggestions. We added more information on the wealth index findings.*

7. • In the conclusion, the authors (supported by the data in the current study) have advocated a focus on people with recently treated STIs as one of the targets for the control of HIV. Just a thought: would enhancement of the prevention of STIs be a more feasible strategy?

***Response:*** *Thank you for this comment. Prevention and treatment of STIs should be prioritized.*

8. • One limitation of the use of spatial autocorrelation is the phenomenon of modifiable areal unit problem (MAUP) whereby different results may be obtained from analysis of the same data, grouped into different sets of areal units. It is not clear if the geographical units for analysis in the various countries were of similar size.

***Response****: Different areal units were used for the countries, and this was stated as part of the limitations.*

Reviewer #2: In this paper, Adetokunboh and Are present an analysis of the most recent DHS data from six Southern African countries. The authors identify several predictors for HIV positive status as well as geographical hotspots for HIV within these countries.

***Response****: Thank you for the comments.*

I have the following comments:
Major:
1) This is essentially a relatively standard statistical analysis of DHS data: the key results of the DHS surveys are publicly available, and the dataset is freely available for researchers to analyse. It is surprising to me that such analysis has not already been done. I propose the authors to first run a systematic literature search to check what studies using this data have already been conducted. The main results are no surprise and in my view it is already common knowledge that factors such as high age or residing in an urban setting are associated with positive HIV status.

***Response:*** *Thank you for the suggestion to do a review. We conducted a rapid review and evidence mapping before the study started. A few studies already used DHS data for some aspects of our research, but those studies focused on individual countries while our own paper is regional and comparing progress over the years. We made references to some of those papers in the background and discussion sections.*

2) The DHS data has a great amount of different variables. How did the authors choose the variables that were included in the analysis? Most variables were such that are already known to some extent correlate with HIV status, so the added value of this study seems limited. If the authors used a systematic approach to select the variables for the analyses (or e.g. followed the results of a previous analysis), this should be described in detail.

***Response****: The determinants were selected based on previous similar studies. We have made references to the papers.*

3) It is a bit unclear why the AIDSinfo prevalence data were included in this analysis. The analysis of time trends seems completely independent of the DHS data? Please note that the AIDSinfo estimates are themselves a result of models that use different data sources, so to me a simple description of such estimates does not count as original research.

***Response:*** *The reason for using both DHS and AIDSInfo data is for triangulation purposes. AIDSInfo data was also included to appreciate the prevalence trend over a period in the southern African region.*

Detailed comments:
4) Abstract, Methods: Please be more specific of the actual analyses that you conducted (multivariable regression analysis of DHS data; autocorrelation analysis; etc).

***Response:*** *Thank you for the suggestion. We have added specific analyses.*

5) Introduction, last sentence: 73% suppression is essentially equivalent with the achievement of the 90-90-90 target, so no need to distinguish

***Response****: Thank you for the comments.*

6) Methods: Please be more clear that you used the DHS data and describe the data according to the usual DHS structure so that it's clear what datasets were used.

*Response: Thank you for the suggestion. We have made the correction.*

7) The type of analysis seems not to be mentioned anywhere. Presumably it was logistic regression?

***Response:*** *It was logistic regression.*

8) Be careful about the terms: an analysis adjusted for several variables is multivariable, not multivariate, and if you report crude odds ratios, these are results of univariable, not multivariable, analysis

***Response:*** *Thank you for the comments.*

9) Under data analysis point d), it is unclear what you mean by “recommended”.

***Response:*** *The statement has been corrected.*

10) Data analysis point e), how is the timing (“for each of the available data years”)? To my understanding only one (the latest) dataset from each country was analysed?

***Response:*** *Thank you. The statement has been corrected.*

11) Results: The paragraph “HIV prevalence trend…” is difficult to understand. The methods for estimating the yearly decrease are not given - did you use some formal statistical method, or is this simply the difference between 2020 and 2011 divided by 10 on the geometric scale? Since the direction in South Africa changed, how is it possible to give an annual increase until 2020? As suggested in the earlier comment, the added value of this analysis is unclear as it’s not related to the DHS data.

***Response****: Thank you for the comments. As we stated earlier, the inclusion of figure 1 was for triangulation purpose and as a supportive step to show how each country did over a decade since we could not access such long-term data for DHS for all the countries. South Africa had average annual increase unlike other countries that had reductions.*

12) In my view, the most interesting results are those of the geospatial analysis. It would be nice to show these figures in the main manuscript.

***Response:*** *Thank you for the suggestions.*

13) Discussion, second paragraph: It seems that prevalence and incidence are mixed up in this paragraph (and for example, it is unclear what “15% new infections in RSA in 2020” actually means). Please note that high prevalence is not necessarily a negative thing: with the expansion of ART coverage, people living with HIV are now living much longer and thus their share of the total population should not decrease too rapidly. The fact that HIV prevalence has continued to increase in RSA over time doesn’t necessarily imply that the epidemic is not under control (although this can of course be one reason). To my understanding the study only reported prevalence, not incidence, data, so nothing certain can be said about the actual epidemic control.

***Response:*** *Thank you for the comments. We have corrected the statement.*

14) Moreover, I strongly disagree that reduction in HIV prevalence would be a result of religious practices. During the study period, all mentioned countries have moved from the limited ART coverage and CD4 dependent thresholds etc towards universal ART access to all PLHIV, and it is well known that successful ART minimized onward transmission. I would argue that the decrease in prevalence (and in particular incidence) is mainly attributable to high ART coverage, increased knowledge, less stigma etc.

***Response:*** *Thank you for the comment.*

15) The language needs some (minor) editing.

***Response:*** *Thank you for the comment. We did a comprehensive editing.*
