# Supplementary material for: Spatial distribution and determinants of HIV high burden in the Southern African sub-region
Source: PLoS One. 2024 Apr 26;19(4):e0301850. doi: 10.1371/journal.pone.0301850 (PMC11051620; doi:10.1371/journal.pone.0301850)
Supplement: Attachment — Submitted filename: Response to reviewers_Spatial Distribution_2023-11-21.docx [file pone.0301850.s004.docx]

**REVIEWER’S COMMENTS AND RESPONSES**

We would like to thank the reviewer for taking the time to provide valuable comments and suggestions. We believe that these insights have significantly improved the quality and presentation of our manuscript. We have addressed all the comments and made the necessary changes to the manuscript. Below, you will find our responses (highlighted in green) and the corresponding modifications to the manuscript (highlighted in yellow).

Reviewer #3: The authors stated, "Combining spatial analysis and the evaluation of the determinants of the HIV burden in Southern African countries is essential for better understanding of the disease dynamics in high burden settings." However, the authors failed to effectively persuade why this combination leads to a qualitative improvement in understanding the disease dynamics. While the authors explained the importance of spatial analysis of HIV infection and acknowledged the limited relevant previous studies in sub-Saharan Africa, this study only added two new countries on it, whereas four countries were already reported in Bulstra et al.'s study published in 2020. The significance of this addition is questionable.
Regarding the determinants of HIV, the unique contribution of this study remains unclear. The authors mentioned that "previous studies were either restricted to just a locality or conducted more than a decade ago." However, there are numerous studies available from other countries, raising concerns about the need to revisit the Southern African region for further research. The authors did not reference previous studies sufficiently, which weakens the justification for their current investigation.
To strengthen the study's rationale, it is crucial for the authors to thoroughly examine and reference previous reports. They must identify sufficient reasons for studying this subject in the Southern African region and clearly articulate the novel insights they aim to bring to the field.

***Response:*** We thank the reviewer for the comments and suggestions, particularly for highlighting the need for a clearer justification. We have added several paragraphs to various sections of the paper to strengthen the report.

The progress in addressing the HIV epidemic is dynamic, and many sub-Saharan African countries have achieved notable advancements in reducing new infections and improving access to treatment and care; however, several Southern African countries are still battling relatively high HIV prevalence rates. This makes the area a region of interest that needs to be thoroughly investigated [2].

A geospatial analytical study characterizing areas with high levels of HIV transmission among seven countries in Eastern and Southern Africa regions shows that, among young adults, there were areas with relatively high prevalence alternating with low prevalence areas, signifying the presence of areas with high levels of HIV transmission [7]. However, this study is limited by the restriction of the participants to only young adults (15–29 years of age) and data that were collected as far back as 2008. Using data obtained from all adult age groups is expected to be more robust and informative than focusing on a single age group, as there are important differences in epidemiology, biology, and behavior across age groups [8]. A study on mapping HIV prevalence in sub-Saharan Africa shows significant local variations in the rate and direction of change in HIV prevalence between 2000 and 2017 in the region [9]. These findings demonstrate how significant local variations can be masked by national-level trends, especially when several important age cohorts are overlooked.

This work contributes to the existing body of knowledge by incorporating two fundamental approaches: geospatial analysis and multivariable logistic regression. By merging spatial pattern analysis with logistic regression, we achieve a more robust and reliable exploration of geographic, HIV, and sociodemographic data. This combined approach enhances precision in identifying hotspots and also pinpoints local drivers of disease dynamics. It leverages the individual strengths of each technique, fostering a more comprehensive understanding of the geospatial distribution of HIV in Southern Africa. These methods serve as a valuable framework in various domains, including public health, resource management, urban planning, and environmental science. It also provides a premise and reference point for future studies aimed at detecting the potential fading, emergence, or re-emergence of HIV hotspots. This, in turn, can guide timely, spatially targeted interventions. The outcomes will inform public health strategies, policy development, resource distribution, and the understanding of trends over time, all of which are crucial for combating the disease and enhancing the lives of those affected by HIV.

1) This study utilized a secondary dataset; therefore, the authors explained the source of the original dataset and the process of extracting the data for their research. It is important to note that the authors did not personally select survey sites or conduct data collection. However, the statements presented in the methods section are somewhat confusing. To improve clarity, further elaboration is required to clearly distinguish the steps involved in obtaining the secondary dataset from the actual data collection procedures, which were not carried out by the authors themselves.

***Response:*** Thank you for your comment. We added statements on how the data were collected and analyzed.

The survey data for this study was a cross-sectional representative sample of households extracted from the Measure DHS website (http://www.dhs program.com) [9]. The sampling frame for the surveys consisted of enumeration areas across each included country. The surveys consisted of a two-stage sample design with standardized questionnaires administered to the participants [9]. Trained interviewers gave questionnaires to selected participants in each of the included countries during the data collection stage. Permission was granted for data extraction by Measure DHS and the data were downloaded in STATA format.

DHS survey samples were selected using a stratified, two-stage cluster design with enumeration areas as the primary sampling unit and the households as the secondary sampling units in each of the participating countries. During the interview, individuals who met the eligibility requirements and were between the ages of 15 and 49 were encouraged to test for HIV voluntarily. After that, the skilled interviewers obtained dried blood spot samples from finger pricks for an HIV test. The anonymous linked procedure serves as the foundation for the sample collection and analysis protocol. The DHS program methodology allows for combining HIV test results with other information gathered based on each respondent's unique questionnaire. The DHS program was funded by various development partners, including the Global Fund to Fight AIDS, Tuberculosis and Malaria, the United States Agency for International Development, etc., and technical assistance from MEASURE DHS, ICF International, Calverton, Maryland, USA. The corresponding National Ministry of Health or Research/Statistical agencies implemented the DHS program.

2) Table 1: The superscript symbol "*", was not explained elsewhere in the document. Please provide a clear explanation of its meaning to avoid any confusion for the readers.

***Response:*** Thank you for the suggestion. A footnote has been added.
* Number of participants tested positive for HIV and percentage in parenthesis.

3) Figure 1: To provide more detailed information, please mark the data points and connect them with lines instead of using continuous lines.

***Response:*** Thank you for your suggestion. The figure has been delected.

4) Table 3 not ‘Table 2’ shows the relationship between socio-demographic factors and determinants.
***Response:*** Thank you for the comment., we have modified it accordingly.

5) S1 Table: This table contains the crude (univariate) results. Therefore, please remove 'multivariate' from the heading and state that the 'adjusted' results mean adjustment for all other predictive variables.

***Responses:*** Thank you for your suggestion. We have added more information about the adjusted results.

6) Please provide successive explanations for Figure S1-S6 to improve readability and help readers understand the content more easily.

***Responses:*** Thank you for your suggestion. We have provided additional explanations. See below.

The study shows a Low-High HIV occurrence and cluster around the Central Region of Malawi; this implies that the area has low HIV prevalence, but its neighboring clusters have high HIV prevalence; the High-Low cluster in Zambezia Province - the area has a high HIV prevalence, but its neighboring areas have low values for the same variable; and the High-High cluster in Maputo Province of Mozambique - the area has a high HIV prevalence, likewise the neighboring clusters. In addition, there were also Low-Low clusters in the Omaheke, Khomas and Hardap Regions of Namibia – the clusters and neighboring clusters has low HIV prevalence; High-High cluster in Southern Province of Zambia; and Low-High cluster in Bulawayo Province and High-Low cluster in Mashonaland East Province of Zimbabwe.

7) Providing a clear image using hotspot cluster windows may offer advantages. However, it is important to note that the information presented is less detailed than that of a usual spatial mapping. To improve the clarity of the analysis, the authors should either provide a persuasive explanation of the advantages of using hotspot cluster windows or consider changing the approach to a usual mapping technique that offers more comprehensive spatial information.

***Response:*** Thank you for your comment. We added statements on hotspot cluster windows.

We used kernel density estimation (KDE) because it can effectively detect clusters of events within a given geographic area. KDE is a hotspot mapping technique that produces a smooth and continuous surface map that illustrates gradients of the variation in event intensity across the study fields without being constrained by theme borders [42]. Its striking visual appeal makes it a popular mapping technique and one of the frequently used approaches for point pattern analysis [42,43]. KDE differs from other mapping techniques in that it uses a weighting function based on a constant bandwidth or search radius to generate a surface based on a nonparametric estimate of the intensity function across cell grids [44]. We can obtain the bandwidth and then determine the smallest distance at which clustering is most intense and significant [42].

8) “This finding contradicts Parkhurst's assertion that the relationship between HIV infection and household wealth index did not show reliable inclinations among Africans [22].” : Parkhurst (2010) stated that “the relationship between the prevalence of HIV infection and household wealth quintile did not show consistent trends in all countries. In particular, rates of HIV infection in higher-income countries did not increase with wealth.” The results of this study, where wealth level was found to be significant in all the countries except Zimbabewe, do not contradict Parkhurst’s report that the relationship is not consistent.

***Response:*** Thank you for your comment. We have rectified the statement.

HIV infection is assumed to be prevalent among the poor and with a lesser number among the middle and upper social class [26]. Gaumer et al. show that wealth effects on HIV prevalence are usually smaller and statistically insignificant; however, a high wealth index was not associated with higher HIV prevalence, while low wealth was associated with a higher risk among certain sections of the population. [27].

9) Conclusion needs revision to show the results and implication of this study.

***Response:*** Thank you for your comment. We have added the following paragraph to the manuscript.

Our findings highlight the significance of regional differences in HIV prevalence as well as the impact of sociodemographic determinants and sexual behaviors. Our results suggest that analysis of subnational data could provide reasonable estimates of the wide-ranging spatial structure of the HIV epidemic in selected Southern African settings. However, similar analyses should be conducted at district and municipality levels to assess community-level patterns. The spatial distribution of high burden areas for HIV in the selected countries was more pronounced in the major cities. Other measures such as prevention and identification of STIs should also be prioritized. Determinants such as individuals who are divorced or widowed, middle-aged women, and people who recently treated STIs, should be the focus of HIV prevention and control interventions in the Southern Africa Region. The findings of this study are useful in developing HIV prevention programs as it identifies the areas and communities that require additional resources and attention. Healthcare policymakers especially in resource-constrained communities in the Southern African region can apply our findings to identify areas to target for the design of interventions to reduce HIV transmission and inform the prevention and control programs. Lastly, HIV control interventions such as HIV/AIDS awareness campaigns, ART services and adherence support for those on ART should be focused on locations identified as hotspot clusters.
