# Supplementary material for: Spatial distribution and determinants of HIV high burden in the Southern African sub-region
Source: PLoS One. 2024 Apr 26;19(4):e0301850. doi: 10.1371/journal.pone.0301850 (PMC11051620; doi:10.1371/journal.pone.0301850)
Supplement: Attachment — Submitted filename: Response to reviewers_Spatial Distribution_2024=02-18.docx [file pone.0301850.s005.docx]

**REVIEWER’S COMMENTS AND RESPONSES	- REVISION 3**

We would like to thank the reviewers for taking the time to provide valuable comments and suggestions. We believe that these insights have significantly improved the quality and presentation of our manuscript. We have addressed all the comments and made the necessary changes to the manuscript.

1. Please remove your figures/ from within your manuscript file, leaving only the individual TIFF/EPS image files. These will be automatically included in the reviewer’s PDF.

***Response:*** *We thank the reviewer for the comments and suggestions. We have removed the figures from the manuscript as suggested.*

2. We note that Figures 1a-1f in your submission contain [map/satellite] images which may be copyrighted. All PLOS content is published under the Creative Commons Attribution License (CC BY 4.0), which means that the manuscript, images, and Supporting Information files will be freely available online, and any third party is permitted to access, download, copy, distribute, and use these materials in any way, even commercially, with proper attribution. For these reasons, we cannot publish previously copyrighted maps or satellite images created using proprietary data, such as Google software (Google Maps, Street View, and Earth). For more information, see our copyright guidelines: http://journals.plos.org/plosone/s/licenses-and-copyright.

We require you to either (1) present written permission from the copyright holder to publish these figures specifically under the CC BY 4.0 license, or (2) remove the figures from your submission:

1. You may seek permission from the original copyright holder of Figures 1a-1f to publish the content specifically under the CC BY 4.0 license.

We recommend that you contact the original copyright holder with the Content Permission Form (http://journals.plos.org/plosone/s/file?id=7c09/content-permission-form.pdf) and the following text:

“I request permission for the open-access journal PLOS ONE to publish XXX under the Creative Commons Attribution License (CCAL) CC BY 4.0 (http://creativecommons.org/licenses/by/4.0/). Please be aware that this license allows unrestricted use and distribution, even commercially, by third parties. Please reply and provide explicit written permission to publish XXX under a CC BY license and complete the attached form.”

Please upload the completed Content Permission Form or other proof of granted permissions as an ""Other"" file with your submission.

In the figure caption of the copyrighted figure, please include the following text: “Reprinted from [ref] under a CC BY license, with permission from [name of publisher], original copyright [original copyright year].”

2. If you are unable to obtain permission from the original copyright holder to publish these figures under the CC BY 4.0 license or if the copyright holder’s requirements are incompatible with the CC BY 4.0 license, please either i) remove the figure or ii) supply a replacement figure that complies with the CC BY 4.0 license. Please check copyright information on all replacement figures and update the figure caption with source information. If applicable, please specify in the figure caption text when a figure is similar but not identical to the original image and is therefore for illustrative purposes only.

The following resources for replacing copyrighted map figures may be helpful:

USGS National Map Viewer (public domain): http://viewer.nationalmap.gov/viewer/

The Gateway to Astronaut Photography of Earth (public domain): http://eol.jsc.nasa.gov/sseop/clickmap/

Maps at the CIA (public domain): https://www.cia.gov/library/publications/the-world-factbook/index.html and https://www.cia.gov/library/publications/cia-maps-publications/index.html

NASA Earth Observatory (public domain): http://earthobservatory.nasa.gov/

Landsat: http://landsat.visibleearth.nasa.gov/

USGS EROS (Earth Resources Observatory and Science (EROS) Center) (public domain): http://eros.usgs.gov/#

Natural Earth (public domain): http://www.naturalearthdata.com/"

***Response:*** *We thank the reviewer for the suggestions. We were unable to get permission from the Measure DHS for the use of the GIS shapefiles and copyrighted maps. However, we used GADM database of Global Administrative Areas v 4.1.*

GADM data are freely available for academic use and other non-commercial uses. Using the data to create maps for publishing academic research articles is allowed. Thus, could use the maps made with GADM data for figures in articles published by PLoS, Springer Nature, Elsevier, MDPI, etc. You are allowed (but not required) to publish these articles (and the maps they contain) under an open license such as CC-BY as is the case with PLoS journals and maybe the case with other open access articles.

3. Thank you for uploading your study's underlying data set. Unfortunately, the repository you have noted in your Data Availability statement does not qualify as an acceptable data repository according to PLOS's standards.

At this time, please upload the minimal data set necessary to replicate your study's findings to a stable, public repository (such as figshare or Dryad) and provide us with the relevant URLs, DOIs, or accession numbers that may be used to access these data. For a list of recommended repositories and additional information on PLOS standards for data deposition, please see <https://journals.plos.org/plosone/s/recommended-repositories>.

***Response:*** *Thank you for the suggestions. We have uploaded the data set on figshare:* [*https://figshare.com/s/33e95ee4594a7c146e3b*](https://figshare.com/s/33e95ee4594a7c146e3b)

**REVIEWER’S COMMENTS AND RESPONSES - REVISION 2**

We would like to thank the reviewer for taking the time to provide valuable comments and suggestions. We believe that these insights have significantly improved the quality and presentation of our manuscript. We have addressed all the comments and made the necessary changes to the manuscript.

Reviewer #3: The revision seems improved. One recommendation was added.
On the Figure 1~Figure 6, please add the year that the data were obtained and rearrange them into from Figure 1 a) to Figure 1 g).

***Response:*** *We thank the reviewer for the comments and suggestions. We have renamed the figures as suggested.*
